# Book Reviews

**Published:** 1892-04

**Authors:** 


### *BOOK REVIEWS.*

---

PRESCRIBERS' PHARMACOPŒIA, A SYNOPSIS OF THE MORE RECENT REMEDIES, OFFICIAL AND UNOFFICIAL, WITH A THERAPEUTIC INDEX. By a Member of the Pharmaceutical Society of Great Britain. Kemp & Co., Bombay, India, 1891.

This is the second and revised edition of a work which first appeared about one year ago, and which was prepared with particular reference to the properties and therapeutics of new drugs, such as aristol, chloralamid, sulphonal, exalgine, etc. In addition there are chapters on Urinalysis, Hypodermic Medication, General Testing, etc. As a rule the preparation has been made according to the British Pharmacopœia, but in many places the preparations of the U. S. P. have been included. The book will be found useful to both pharmacist and physician. It has been prepared with great care, and makes a very serviceable little volume for ready reference.

---

TREATISE ON DISEASES OF THE EAR. By D. B. St. John Roosa, M. D., Professor of Diseases of the Eye and Ear in the New York Post-Graduate Medical School and Hospital, etc. New York. William Wood & Co.

The popularity of this work is well attested by the fact of its having now reached its seventh edition. As a writer and teacher Dr. Roosa is well known for his ability, and this work of his is pervaded throughout with that charm of presentation so characteristic of the man. His work is now one of the best text-books in the English language upon the diseases of the ear, and as such it is deservedly popular. The author makes the book a good one for reference in that the views of other distinguished aurists are given whenever a subject of importance is discussed. He believes that the operative interference for the relief of deafness has not been sufficiently tested for the expression of a decided opinion *pro* and *con*, but fully believes in it when chronic suppurative catarrh of the middle ear is dependent upon a necrosed condition of the ossicles. The book will prove a valuable addition to any library. C. D. R.

---

PRACTICAL TREATISE ON ELECTRICITY IN GYNÆCOLOGY. By Egbert H. Grandin, M. D., Obstetric Surgeon, New York Maternity Hospital; and Josephus H. Gunning, M. D., Instructor in Electro-Therapeutics, New York Post-Graduate Medical School and Hospital. Illustrated. New York: William Wood & Company.

This is a very convenient and serviceable description of the uses of electricity in gynæcology. Contents: General Considerations and Description of Apparatus; Routine Uses of Electricity; Electrolysis; Static, or Frictional Electricity; The Treatment of Malignant Growths by the Galvano-Cautery; and Electricity in Obstetrics. The authors do not make a "hobby" of electricity, and their views have therefore been presented conscientiously and without bias. They advise the use of electrolysis for uterine fibroids before the more heroic surgical operations.

In extra-uterine pregnancy they recommend its employment as a means of destroying the fœtus, and support their views with such high authority as Thomas, Mann and others. The work is safe and conservative, and will be found useful by those in need of a work on therapeutic electricity.

---

HISTORY OF CIRCUMCISION FROM THE EARLIEST TIMES TO THE PRESENT. By P. C. Remondino, M. D., Philadelphia. F. A. Davis.

This is not a strictly technical work, and indeed the author does not intend it as such. The author holds that the prepuce, along with the climbing muscle and the appendix-vermiformis is an evolutionary remnant of our simian and arboreal forebears, and that it must succumb to the advance of civilization.

It has no function other than a mischievous one. Therefore he advocates universal circumcision.

Not only shall the offending preputial appendage be removed from the hapless individual too lavishly endowed, but also "from him that hath not shall be taken away even that which he hath." *Circumcision a outrance!* That these extreme ideas will gain general acceptance is hardly possible. His argument is ingenious and plausible, but scarcely *ad hominem.* The book is valuable as a very readable compilation of historical facts relating to circumcision and kindred subjects, as eunuchism, infibulation, muzzling and other curious and obsolete practices, but apart from the general interest attached to these, it is of uncertain utility.

---

PRELIMINARY PROGRAMME FORTY-THIRD ANNUAL SESSION OF THE MEDICAL ASSOCIATION OF GEORGIA, TO BE HELD IN COLUMBUS, GA., APRIL 20TH, 21ST AND 22D, 1892.—*Papers to be Read:* Partial list. The President's Annual Address, G. W. Mulligan, M. D., Washington; Cough—Some of its Causes and Treatment, Chas. D. Roy, A. B., M. D., Atlanta; So-Called Typho-Malarial Fever, W. P. Williams, M. D., Waycross; Preliminary Observations on the Behavior of Iodine in the Presence of Camphor, Menthol, Thymol, etc., R. J. Nunn, M. D., Savannah; Some Observations upon Cataract Operations and After Treatment, A. W. Calhoun, M. D., Atlanta; Remittent Fever, A. C. Blain, M. D., Brunswick; Extirpation of Rectum for Carcinoma, J. McF. Gaston, M. D., Atlanta; Some of the Fads and Fancies of the Medical Profession, J. C. LeHardy, M. D., Savannah; Treatment of Pneumonia, with Report of Cases, H. Perdue, M. D., Barnesville; How Shall We Manage the

